# Ameliorating Impact of Prophylactic Intranasal Oxytocin on Signs of Fear in a Rat Model of Traumatic Stress

**DOI:** 10.3389/fnbeh.2018.00105

**Published:** 2018-05-28

**Authors:** Micah D. Renicker, Nicholas Cysewski, Samuel Palmer, Dmytro Nakonechnyy, Andrew Keef, Morgan Thomas, Krisztian Magori, David P. Daberkow

**Affiliations:** Department of Biology, Eastern Washington University, Cheney, WA, United States

**Keywords:** oxytocin, intranasal, stress, fear, PTSD

## Abstract

Oxytocin treatment reduces signs of long-term emotional stress after exposure to trauma; however, little is known about the potential protective effects of oxytocin treatment on behavioral and physiological changes associated with extreme stress exposure. The objective of this study was to investigate oxytocin treatment as a prophylactic measure against rat signs of fear. Two separate experiments were conducted in which the time of intranasal oxytocin administration differed. Intranasal oxytocin (1.0 μg/kg) was administered 5 min after daily exposure to foot shock in Experiment #1 and 1 h before foot shock in Experiment #2. In Experiment #1, possible massage-evoked oxytocin release (5 min after foot shock) was also investigated. In both experiments, a contextual fear conditioning procedure was employed in which stress was induced via inescapable foot shock (3 days, 40 shocks/day, 8 mA/shock) in a fear conditioning chamber. Male Sprague-Dawley rats (*n* = 24) were divided into four groups (*n* = 6, per group) for each experiment. Experiment #1 groups: *Control Exp#1* (intranasal saline and no foot shock); *Stress Exp#1* (intranasal saline 5 min after foot shock); *Massage+Stress Exp#1* (massage-like stroking and intranasal saline 5 min after foot shock); *Oxytocin+Stress Exp#1* (intranasal oxytocin 5 min after foot shock). Experiment #2 groups: *Control Exp#2* (intranasal saline and no foot shock); *Stress Exp#2* (intranasal saline 1 h before foot shock); *Oxytocin Exp#2* (intranasal oxytocin and no foot shock); *Oxytocin+Stress Exp#2* (intranasal oxytocin 1 h before foot shock). One week after fear conditioning (and other treatments), rats were independently evaluated for behavioral signs of fear. Two weeks after conditioning, physiological signs of fear were also assessed (Experiment #1). Relative to controls, rats treated with intranasal oxytocin 5 min after daily foot shock sessions exhibited significantly less immobility upon re-exposure to the shock chamber and attenuated physiological responses related to fear (e.g., elevated heart rate and blood pressure). Furthermore, intranasal oxytocin treatment given 1 h before daily foot shock sessions significantly decreased immobility and defecation upon re-exposure to the shock chamber, relative to controls. The results of this study suggest that prophylactic intranasal oxytocin, administered contemporaneously with aversive stimuli, mitigates behavioral and physiological responses associated with traumatic stress.

## Introduction

Oxytocin is a neurohormone produced by neurons of the paraventricular and supraoptic nuclei in the hypothalamus. It influences a variety of social and reproductive behaviors (Donaldson and Young, 2008; Lee et al., 2009; Campbell, 2010; Meyer-Lindenberg et al., 2011; Love, 2014). Administered after traumatization, oxytocin reduces signs associated with stress in human clinical trials (Fischer-Shofty et al., 2010; Olff et al., 2010, 2013; Acheson et al., 2013; Bakermans-Kranenburg and van IJzendoorn, 2013; Stevens et al., 2013; Koch et al., 2014; MacDonald and Feifel, 2014; Nawijn et al., 2016; Sack et al., 2017), as well as in rodent studies (Missig et al., 2010; Ayers et al., 2011; Eskandarian et al., 2013; Zoicas et al., 2014; Janezic et al., 2016). However, the efficacy of oxytocin as a prophylactic, or preventive, treatment on lasting signs of stress has been less explored in humans (Frijling et al., 2014; Ostrowski and Delahanty, 2014; van Zuiden et al., 2017) and there is a dearth of related research with rodents.

Intranasal administration of oxytocin is an effective therapeutic delivery method in humans (Guastella et al., 2010; Acheson et al., 2013; Veening and Olivier, 2013; Nawijn et al., 2016; Sack et al., 2017; van Zuiden et al., 2017). Intranasal oxytocin administration has also been shown to impact specific brain areas considered “social” regions (Bethlehem et al., 2013). While the mechanisms are not clearly understood, intranasal oxytocin administration increases oxytocin levels in the cerebrospinal fluid (Chang et al., 2012; Neumann et al., 2013; Striepens et al., 2013). Oxytocin is a small peptide of nine amino acids (a nonapeptide) and it has been suggested that it may partially pass through the blood brain barrier (Ermisch et al., 1985). However, oxytocin administered intranasally is thought to bypass the blood brain barrier (Talegaonkar and Mishra, 2004; Wu et al., 2008) and therefore produces higher levels of oxytocin in the brain than can be accomplished via peripheral administration (Neumann et al., 2013). Research suggesting that intranasal administration of oxytocin directly and effectively impacts brain function warrants further investigation into its potential therapeutic effects, including any effects on conditions produced by exposure to extreme stress.

Stress alters the normal physiological equilibrium. Chronic foot shock conditioning in rodents induces lasting alterations in behaviors relating to fear and threat of aversive stimuli (LeDoux, 2000, 2014). The hallmark of these stress-induced behavioral changes is an increase in motionless periods (“freezing”) upon re-exposure to the context in which the stressful stimuli were delivered, the foot shock chamber (Sahraei et al., 2012; Yu et al., 2012; Gao et al., 2014). Another sign of fear in rodents is increased defecation when re-exposed to stress-inducing stimuli (Lester, 1968; Stam et al., 1995; Gao et al., 2011). Increased defecation (“colonic motility”) is a measure highly sensitive to psychological stress in rats (Verleye and Gillardin, 2004). Furthermore, many studies have demonstrated fear-related changes in cardiovascular function in rodents (Young and Leaton, 1994; Baudrie et al., 1997; Zhang et al., 2004; Liu et al., 2013, 2014). The purpose of this study was to investigate the effects of oxytocin treatment as a preventive measure against behavioral (e.g., freezing and defecation) and physiological (e.g., resting heart rate and blood pressure) signs of fear in a rat model of traumatic stress. The hypothesis is that prophylactic oxytocin treatment, administered 5 min after (Experiment #1) or 1 h before (Experiment #2) contextual fear conditioning, attenuates behavioral and physiological signs of fear in the rat.

## Materials and Methods

### Experiment #1—Animals

Sprague-Dawley rats (male, 4–5 months, ~400 g) were divided into four groups (*n* = 6, per group). Experiment #1 groups: *Control Exp#1* (intranasal saline administration and no foot shock); *Stress Exp#1* (intranasal saline administration 5 min after foot shock); *Massage+Stress Exp#1* (massage-like stroking and intranasal saline administration 5 min after foot shock); *Oxytocin+Stress Exp#1* (intranasal oxytocin administration 5 min after foot shock). Rats were housed (two per cage, according to groups) with free access to food/water and kept on a 12 h alternating light/dark schedule. All rats were conditioned to procedural human handling for 1.5 weeks prior to the treatments and fear conditioning.

### Experiment #1—Fear Conditioning

Signs of fear were induced via a contextual fear conditioning paradigm employing inescapable foot shocks (Sahraei et al., 2012; Yu et al., 2012; Gao et al., 2014; Janezic et al., 2016). The foot shock was administered in typical fear conditioning shock chamber (the “context”), a closed Plexiglas^®^ box (1 ft × 1 ft) with a metal grate floor. The metal floor was connected to a transformer dial which controlled the intensity of the electric shock administered to the rat through the metal floor. Electrical shocks (8 mA intensity) were administered 20 times per session, with a duration of 3 s per shock, at various time intervals ranging from 7.5 s to 15 s with an approximate mean interval of 10 s. Rat groups receiving foot shock were subjected to two such fear conditioning sessions per day, morning and afternoon (at 3–4 h intervals), for three consecutive days. The rat groups not receiving foot shock were placed in the fear condition chamber for the same time durations on the same days, but without administration of electric foot shock.

### Experiment #1—Oxytocin Treatments

Intranasal oxytocin (VetOne^®^), or massage treatment, was administered during the 3 days of the fear conditioning procedure. *Oxytocin+Stress Exp#1* received intranasal administration of oxytocin (1.0 μg/kg; Ayers et al., 2011; Neumann et al., 2013) 5 min following the last foot shock on each day of the 3 days. Rat groups not treated with oxytocin received an equivalent intranasally administered volume (μL) of saline. Prior to oxytocin (or saline) administration, rats were lightly anesthetized with isoflurane (4 min) to briefly sedate them and allow for efficient intranasal administration of the oxytocin (or saline). Once anesthetized, rats were cradled in a supine position and administered oxytocin (1.0 μg/kg), or an equivalent volume of saline, within 1 min. The exact administration volume was determined each day according to individual rat weight, but the approximate total administration volume was ~7.5 μL, of which half (~3.75 μL) was administered into each nostril via micropipette. Immediately following the intranasal saline administration, *Massage+Stress Exp#1* underwent a massage procedure (all other rats were returned to their home cages). Before returning to their home cages, *Massage+Stress Exp#1* rats were cradled across the scapula and neck region while subjected to massage-like stroking, which consisted of 5 min of light-pressure, abdominal stroking at approximately 20 cm/s (Lund et al., 2002).

### Experiment #1—Behavioral Measures

Rats were individually placed back into the foot shock chamber (the “context”) for 3 min, but not shocked, to record fear-related behaviors 7 days after contextual fear conditioning. Fear memory in rats is often expressed and measured by an increase in freezing behavior (motionless periods defined as the absence of all movements except for those related to respiration; Gao et al., 2014) when reintroduced to the foot shock chamber. Freezing behavior was recorded on video for later analysis. The foot shock chamber was thoroughly cleaned (Oxivir^®^ disinfectant) after each re-exposure.

To assess anxiety, rats were individually placed in an elevated zero maze for 5 min 11 days after fear conditioning. The elevated zero maze consisted of an annular platform elevated 65 cm above the floor (105 cm in diameter, 10 cm in width). The platform of the elevated zero maze was divided into four segments: two opposing open segments with no walls, and two opposing closed segments with walls extending 27 cm above the platform surface. Each rat was placed in one of the open segments of the zero maze and exploratory behavior was recorded on video for later analysis. The amount of time spent in the open segments of the zero maze was measured, which is suggested to be inversely related to the anxiety level of the animal, as proposed by Shepherd et al. (1994).

### Experiment #1—Physiological Measures

Heart rates and hematocrit levels were recorded 14 days after fear conditioning. Rats were cradled and heart rates were measured using a PowerLab 4/20T with LabChart 5 data recording software (ADInstruments). Hematocrit levels were also assessed because there is a relationship between chronically elevated blood pressure and elevated hematocrit levels (Mandal et al., 1993; Milanovic et al., 2012). Though indirect, this provides a physiological measure of blood pressure that is both easier to obtain and potentially more reliable than cumbersome blood pressure monitoring equipment typically designed to attach to an individual rat’s tail. Hematocrit levels were measured from blood samples obtained by pricking the tip of the rats’ tails and collecting blood in microcapillary tubes, which were subsequently centrifuged for 60 s (Mandal et al., 1993; Abatan et al., 2008; Milanovic et al., 2012).

### Experiment #2—Animals

Sprague-Dawley rats (male, 2–3 months, ~250 g) were divided into 4 groups (*n* = 6, per group). Experiment #2 groups: *Control Exp#2* (intranasal saline administration and no foot shock); *Stress Exp#2* (intranasal saline administration 1 h before foot shock); *Oxytocin Exp#2* (intranasal oxytocin administration and no foot shock); *Oxytocin+Stress Exp#2* (oxytocin administration 1 h before foot-shock). All rats were housed (2 per cage, according to groups) with free access to food/water and kept on a 12 h alternating light/dark schedule. Rats were conditioned to procedural human handling for 2–3 weeks prior to the treatments and fear conditioning.

### Experiment #2—Fear Conditioning

Signs of fear were induced via the same 3-day contextual fear conditioning procedure employed in Experiment #1; however, in Experiment #2, the first foot shock session was conducted 1 h after intranasal oxytocin administration to investigate the impact of pretreatment on signs of fear.

### Experiment #2—Oxytocin Treatment

One hour prior to fear conditioning, the rats were lightly anesthetized and treated with intranasal oxytocin (1.0 μg/kg), or saline, as described in Experiment #1. Massage was not investigated in Experiment #2; instead, a group was added in which the rats were treated with intranasal oxytocin without exposure to foot shock (*Oxytocin Exp#2*).

### Experiment #2—Behavioral Measures

Seven days after fear conditioning (and other treatments), motionless periods (freezing) upon re-exposure to the foot shock chamber was assessed as described in Experiment #1, except the rats were re-exposed to the foot shock chamber for 5 min (2 min longer than the 3 min re-exposure time used in Experiment #1). Additionally, defecation upon re-exposure to the foot shock chamber was assessed in Experiment #2. After each re-exposure to the foot shock chamber, the rat’s fecal production was collected and weighed. Subsequently, the foot shock chamber was thoroughly cleaned (Oxivir^®^ disinfectant).

### Statistical Analysis

Statistical analysis on the data from Experiments #1 and #2 was performed using R software (version 3.2.3) for Windows. In Experiment #1, all data were analyzed with a one-way analysis of variance (ANOVA) followed by a *post hoc* Tukey’s HSD test for multiple comparisons. Student’s *t*-tests were also employed on the zero maze and heart rate data to further evaluate specific differences between groups for these measures. In Experiment #2, all data were analyzed with a two-way ANOVA followed by a *post hoc* Tukey’s HSD test for multiple comparisons.

### Ethics Statement

All experimental procedures were approved by Eastern Washington University’s *Institutional Animal Care and Use Committee* (IACUC, protocols #2015-01-06 and #2016-05-01) in accordance with the recommendations of the PHS Policy on Humane Care and Use of Laboratory Animals and the Animal Welfare Act.

## Results

**Figure 1:**
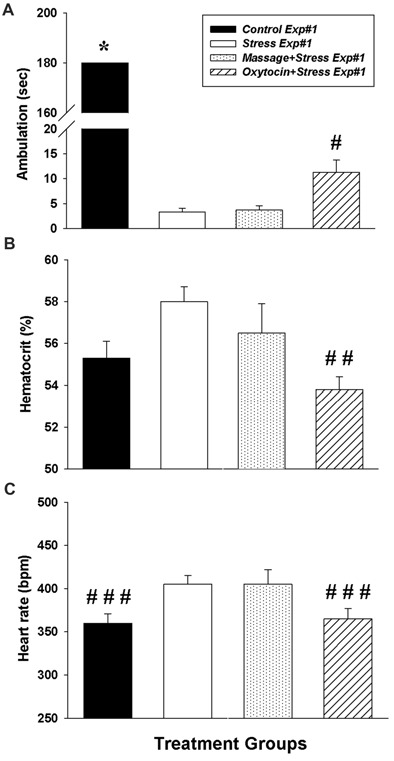
Impact of intranasal oxytocin administered 5 min after (Experiment #1) daily foot shock sessions (3 days, 40 shocks/day, 8 mA/shock). **(A)** Ambulation (inverse of freezing behavior) during 3 min re-exposure to the fear conditioning chamber 7 days after the last day of foot shock. **(B)** Hematocrit levels (indirect assessment of blood pressure) taken 14 days after the last day of foot shock. **(C)** Resting heart rate monitored 14 days after the last day of foot shock. Data analyzed by one-way analysis of variance (ANOVA) followed by *post hoc* Tukey’s HSD test for between-group comparisons (Student’s *t*-tests were used to further assess between-group comparisons of heart rates). *Significantly different than *Stress Exp#1*, Massage+Stress Exp#1 and *Oxytocin+Stress Exp#1* (*p* < 0.05). ^#^Significantly different than *Control Exp#1*, Stress Exp#1 and *Massage+Stress Exp#1* (*p* < 0.05). ^##^Significantly different than *Stress Exp#1* (*p* < 0.05). ^###^Significantly different than *Stress Exp#1* (*p* < 0.05, *t*-test). Bars are means ± SEM (note, all *Control Exp#1* rats were ambulatory the entire 180 s of testing).

In Experiment #1, intranasal oxytocin treatment given 5 min after foot shock significantly reduced behavioral signs of fear. One-way ANOVA revealed an overall significant effect on ambulation (inverse of freezing behavior) (*F*_(3,20)_ = 4191.1, *p* < 0.0001). Tukey’s *post hoc* comparisons revealed significant differences in freezing time between groups (Figure 1A): *Control Exp#1* demonstrated significantly more ambulation (less freezing) vs. *Stress* *Exp#1* (*p* < 0.01), *Massage+Stress* *Exp#1* (*p* < 0.01) and *Oxytocin+Stress* *Exp#1* (*p* < 0.01). Interestingly, there was significantly more ambulation in *Oxytocin+Stress* *Exp#1* vs. *Stress* *Exp#1* (*p* < 0.01) suggesting that prophylactic intranasal oxytocin treatment alleviates the stress-induced freezing response.

Anxiety was also assessed in Experiment #1. The one-way ANOVA on measures of anxiety (time spent in the open areas of the zero maze) revealed overall significance (*F*_(3,20)_ = 3.27, *p* = 0.043). Tukey’s *post hoc* comparisons between groups did not reveal significant differences; therefore, unpaired Student’s *t*-tests were used, which revealed significant differences in measures of anxiety between groups. *Oxytocin+Stress Exp#1* spent significantly more time in the open segments of the zero maze vs. *Control Exp#1* (*p* = 0.046) and *Stress Exp#1* (*p* = 0.041). Unfortunately, *Control Exp#1* did not exhibit the expected high level of exploratory behavior into the open segments, possibly due to temporary sound pollution from the adjacent room (exclusively present during *Control Exp#1* testing); because of the uncontrolled variable (sound pollution) during this phase of zero maze data collection, the zero maze data were deemed inconclusive (data not shown).

In addition to intranasal oxytocin’s significant impact on freezing behavior related to fear, it also had significant effects on physiological signs of fear investigated in Experiment #1. Oxytocin attenuated the stress-induced elevation in hematocrit (indirect measure of blood pressure, Mandal et al., 1993; Milanovic et al., 2012) and heart rate. The one-way ANOVA revealed an overall significant difference in hematocrit (*F*_(3,20)_ = 3.54, *p* = 0.033) and heart rates (*F*_(3,20)_ = 3637.5, *p* < 0.0001). Tukey’s *post hoc* comparisons revealed significant differences in hematocrit (Figure 1B). Interestingly, hematocrit values were significantly lower in *Oxytocin+Stress Exp#1* vs. *Stress Exp#1* (*p* < 0.05). Tukey’s *post hoc* comparisons did not reveal significant differences in heart rates between groups; therefore, unpaired Student’s *t*-tests were used (Figure 1C) which revealed that heart rate was significantly lower in *Control Exp#1* vs. *Stress Exp#1* (*p* = 0.013). Furthermore, Student’s *t*-test revealed heart rate was significantly lower in *Oxytocin+Stress Exp#1* vs. *Stress Exp#1* (*p* = 0.030). Overall, these data suggest that intranasal oxytocin treatment prophylactically alleviates physiological signs of fear.

**Figure 2:**
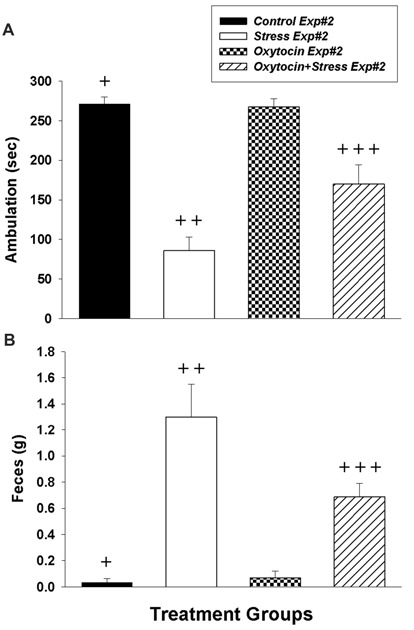
Impact of intranasal oxytocin administered 1 h before (Experiment #2) daily foot shock sessions (3 days, 40 shocks/day, 8 mA/shock). **(A)** Ambulation (inverse of freezing behavior) during 5 min re-exposure to the fear conditioning chamber 7 days after the last day of foot shock. **(B)** Fecal production (colonic motility) during the same 5 min re-exposure. Data analyzed by two-way ANOVA followed by *post hoc* Tukey’s HSD test for between-group comparisons. ^+^Significantly different than *Stress Exp#2* and *Oxytocin+Stress Exp#2* (*p* < 0.05). ^++^Significantly different than *Control Exp#*2, Oxytocin Exp#2 and *Oxytocin+Stress Exp#2* (*p* < 0.05). ^+++^Significantly different than *Stress Exp#*2, Oxytocin Exp#2 and *Oxytocin+Stress Exp#2* (*p* < 0.05). Bars are means ± SEM.

In Experiment #2, intranasal oxytocin pretreatment (1 h prior to shock) significantly diminished the freezing behavior of stressed rats. The two-way ANOVA revealed that stress had a significant effect on ambulation (*F*_(1,20)_ = 74.97, *p* < 0.0001), oxytocin also had a significant effect on ambulation (*F*_(1,20)_ = 6.02, *p* = 0.023), and there was a significant interaction between stress and oxytocin (*F*_(1,20)_ = 7.23, *p* = 0.014). Tukey’s *post hoc* comparisons revealed significant differences in ambulation between groups (Figure 2A): *Control Exp#2* demonstrated significantly more ambulation (less freezing) vs. *Stress* *Exp#2* (*p* < 0.0001) and *Oxytocin+Stress* *Exp#2* (*p* < 0.002). Moreover, in corroboration with Experiment #1, there was significantly more ambulation in *Oxytocin+Stress* *Exp#2* vs. *Stress* *Exp#2* (*p* < 0.008), further substantiating the mitigating effects of prophylactic intranasal oxytocin treatment on freezing behavior.

In addition to the prophylactic effects of oxytocin on freezing behavior, it also had a significant impact on stress-induced defecation, investigated in Experiment #2. The two-way ANOVA revealed that stress had a significant effect on fecal production (*F*_(1,20)_ = 45.88, *p* < 0.0001), oxytocin’s effect on fecal production was close to significance (*F*_(1,20)_ = 4.27, *p* = 0.052), and there was a significant interaction between stress and oxytocin (*F*_(1,20)_ = 5.42, *p* = 0.031). Tukey’s *post hoc* comparisons between groups revealed significant differences in fecal production (Figure 2B): *Control Exp#2* demonstrated significantly less fecal production vs. *Stress* *Exp#2* (*p* < 0.0001) and *Oxytocin+Stress* *Exp#2* (*p* < 0.016). Consistent with oxytocin’s effect of decreasing the freezing behavior of stressed rats, there was significantly less fecal production in *Oxytocin+Stress* *Exp#2* vs. *Stress* *Exp#2* (*p* < 0.026).

## Discussion

### Rodent Model of Traumatic Stress

Modeling neuropsychiatric conditions, such as post-traumatic stress disorder (PTSD), is challenging (reviewed in Deslauriers et al., 2018). Resulting from exposure to psychologically traumatizing events, PTSD is characterized by a constellation of symptoms including intrusion, avoidance, alterations in mood and hyperarousal (American Psychiatric Association, 2013). Many different types of “traumatic stressors” are thought to induce PTSD in humans (Stein et al., 2014); in rodents, however, a common model of “traumatic stress” is based on the chronic electric foot shock fear conditioning paradigm (Sahraei et al., 2012; Yu et al., 2012; Gao et al., 2014; Janezic et al., 2016). Foot shock is the most common aversive stressor used in rodent fear models (Bali and Jaggi, 2015; Flandreau and Toth, 2017; Deslauriers et al., 2018). Although foot shock is not considered ethologically valid, as foot shock does not underlie the development of psychological disorders in humans, it is a physical stressor with emotional components (Bali and Jaggi, 2015). Chronic foot shock conditioning induces lasting alterations in behaviors related to stress and the emotion of fear; however, there is risk in anthropomorphizing rodent models of fear into the conceptualization of human disorders such as PTSD (LeDoux, 2014). That being said, the neural circuits and cellular/molecular mechanisms underlying the acquisition and expression of the conditioned fear response have been characterized and are conserved among mammals (Fanselow and Poulos, 2005; Maren, 2005; Johansen et al., 2011), suggesting mechanistic validity of the model. Pharmacological studies have demonstrated that fluoxetine (Siegmund and Wotjak, 2007) and paroxetine (Shimizu et al., 2004) ameliorate signs of stress in rodents, suggesting predictive validity. Furthermore, some of the core features of PTSD are present after foot shock conditioning (e.g., sleep disturbances, cognitive deficits and avoidance behavior after the traumatic event), suggesting face validity (Bali and Jaggi, 2015). Therefore, the attenuating effects of prophylactic intranasal oxytocin on signs of fear in the rat, demonstrated in this study, may have implications for certain psychiatric conditions such as PTSD.

### Fear Neurocircuitry and Oxytocin

Oxytocin is a neuropeptide that directly interacts with oxytocin receptors in specific parts of the central nervous system; as such, it is considered a neuromodulator in brain regions associated with fear, aggression and social behaviors (Heinrichs and Domes, 2008; Febo and Ferris, 2014). Oxytocin receptors are expressed in the amygdala, a brain area involved in the processing of emotion and cognition (reviewed in Phelps, 2006). Furthermore, oxytocin receptors are also found in reward processing areas of the brain (Febo and Ferris, 2014), including the ventral tegmental area and the nucleus accumbens (Wise and Bozarth, 1987; Nicola, 2016). Hypothalamic oxytocin neurons have direct axonal connections to the amygdala and nucleus accumbens, and are thought to directly modulate the activity of these brain regions (Meyer-Lindenberg et al., 2011; Knobloch et al., 2012; Bethlehem et al., 2013). This circuitry provides a neurobiological pathway through which oxytocin, intranasally delivered to the brain, may alter the acquisition and expression of the behavioral signs of fear observed in this study. Although intranasal oxytocin significantly reduced fear-related behaviors (freezing and defecation), the fact that intranasal oxytocin treatment did not return these response variables to baseline levels (Figures 1A, 2A,B) suggests that, at the dosage administered in this study, oxytocin is providing partial protection from the development of these fear signs and does not entirely inhibit the manifestation of fear.

### HPA Axis and Oxytocin

In addition to its direct effects on brain regions associated with the processing of emotions and motivation, oxytocin also interacts with the hypothalamic-pituitary-adrenal (HPA) axis. This endocrine feedback system is implicated in both stress reactions and the regulation of many body processes (Bhatnagar et al., 2006; Stranahan et al., 2008; Hall et al., 2012; Daskalakis et al., 2013). Oxytocin has been shown to inhibit certain stress responses associated with the HPA axis, such as corticosterone release (Windle et al., 1997; Heinrichs et al., 2003; de Kloet et al., 2006). Oxytocin levels rise following stressful incidents, and higher oxytocin levels correspond to faster recovery from stress related symptoms (Engert et al., 2016). Therefore, prophylactic intranasal oxytocin treatment could potentially provide protection during stressful events via the HPA axis.

### Massage and Oxytocin

Massage has been suggested to induce oxytocin release. Massage treatment in humans has been shown to increase salivary oxytocin levels (Tsuji et al., 2015) and blood oxytocin concentrations (Morhenn et al., 2012; Rapaport et al., 2012). Although massage-like stroking of rats has an anti-nociceptive effect (Agren et al., 1995; Lund et al., 2002) and decreases blood pressure (Kurosawa et al., 1995) via oxytocinergic mechanisms, the massage treatment employed in this study was not effective in reducing fear signs. Therefore, the impact of massage-evoked oxytocin on alleviating signs of fear requires further investigation.

### Oxytocin Prophylaxis

To our knowledge, this is the first study demonstrating that prophylactic intranasal oxytocin, administered soon (minutes) after or before aversive stimuli, attenuates signs of fear in the rat. Intranasal oxytocin given 5 min after daily foot shock significantly attenuated stress-induced freezing behavior and pathological increases in measures of cardiovascular function. Furthermore, intranasal oxytocin treatment 1 h prior to foot shock also attenuated stress-induced freezing behavior and colonic motility. Collectively these data support the hypothesis that prophylactic, or preventative, intranasal oxytocin treatment mitigates behavioral and physiological signs of fear in the rat. Various pharmaceutical and psychosocial interventions have previously been studied as potential preventive treatments for stress-related disorders in humans, such as PTSD (reviewed in Baker et al., 2009; Daskalakis et al., 2013). Most preventive measures that have been explored fall into the category of “early intervention”, in which treatment is given after the traumatic event, but prior to development of related symptoms (reviewed in Birur et al., 2017). Prophylactic oxytocin may offer the same neuroprotective benefits of other treatments (Vaiva et al., 2003; Baker et al., 2009; Daskalakis et al., 2013; Morena et al., 2017) without the risks associated with long-term treatment (Baker et al., 2009). Remedial treatment after a traumatic event might be most practical, as the majority of traumatic events cannot be predicted; however, if prophylactic or contemporaneous treatments offer significant protection, these treatments could be beneficial in settings such as combat warfare, law enforcement operations and preoperative care.

## Author Contributions

MR and NC conceived the project idea. All authors contributed to the project development, data collection and analysis. KM assisted with the statistical analysis. DD supervised and facilitated the research. MR drafted the manuscript, with substantial contributions from DD and MT.

## Conflict of Interest Statement

The authors declare that the research was conducted in the absence of any commercial or financial relationships that could be construed as a potential conflict of interest.
